# Supplementary material for: Interpretable and integrative analysis of single-cell multiomics with scMKL
Source: Commun Biol. 2025 Aug 6;8:1160. doi: 10.1038/s42003-025-08533-7 (PMC12328712; doi:10.1038/s42003-025-08533-7)
Supplement: Supplementary file 1 — Supplementary Information [file 42003_2025_8533_MOESM1_ESM.pdf]

Supplemental Material for Interpretable and Integrative Analysis of Single-Cell Multiomics with scMKL

Samuel D. Kupp[1], Ian A. VanGordon Jr.[1], Mehmet Gönen[2,3], Sadık Esener[1,4], S. Ece Eksi[1,4,5], and Çiğdem Ak*[1,4]

[1] *Cancer Early Detection Advanced Research (CEDAR), Knight Cancer Institute, OHSU, Portland, OR, 97239, USA*

[2] *Department of Industrial Engineering, College of Engineering, Koç University, İstanbul, Türkiye*

[3] *School of Medicine, Koç University, İstanbul, Türkiye*

[4] *Department of Biomedical Engineering, School of Medicine, OHSU, Portland, OR, 97209, USA*

[5] *Division of Oncological Sciences, Knight Cancer Institute, OHSU, Portland, OR, 97239, USA*

*Corresponding Author: ak@ohsu.edu

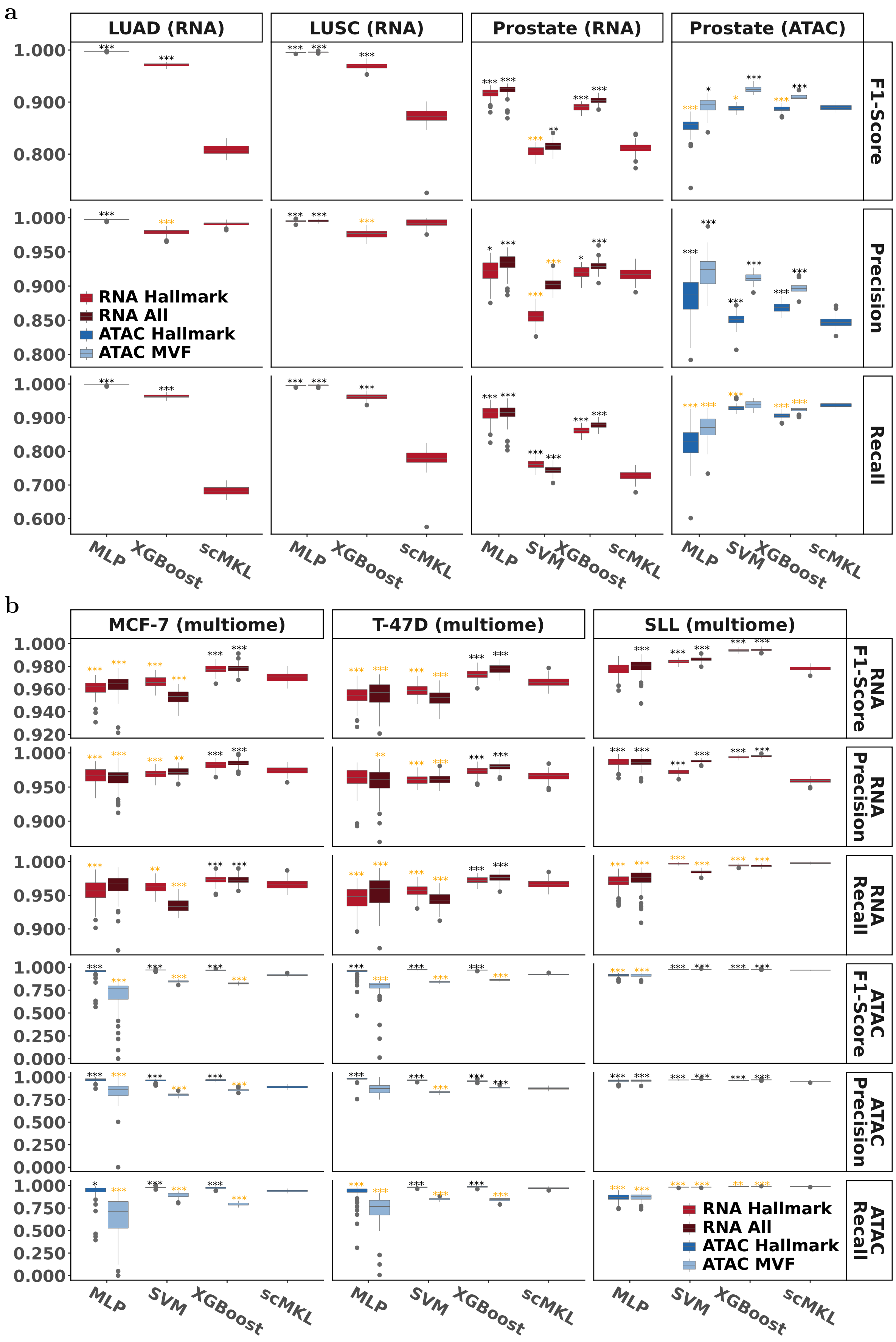
a
LUAD (RNA)
LUSC (RNA)
Prostate (RNA)
Prostate (ATAC)
F1-Score
Precision
Recall
RNA Hallmark
RNA All
ATAC Hallmark
ATAC MVF
MLP
SVM
XGBoost
scMKL
b
MCF-7 (multiome)
T-47D (multiome)
SLL (multiome)
RNA F1-Score
RNA Precision
RNA Recall
ATAC F1-Score
ATAC Precision
ATAC Recall

Figure S1. scMKL provides scalable, flexible, and accurate predictions across diverse datasets.

a-b. Classification performance comparison: scMKL, MLP, XGBoost, and SVM were evaluated across seven datasets, including three multimodal datasets–MCF-7, T-47D, and SLL (panel b) and three unimodal datasets–PCa, LUAD, and LUSC scRNA-seq and PCa scATAC-seq (panel a). Darker color shades represent models trained on all features, while lighter shades denote models using Hallmark or MVF features for RNA and ATAC. *, **, *** are used to denote statistical significance from Wilcoxon test p values < 0.05, 0.01, and 0.001 respectively. Gold indicates scMKL had significantly better performance; black indicates the benchmark algorithm had significantly higher performance.

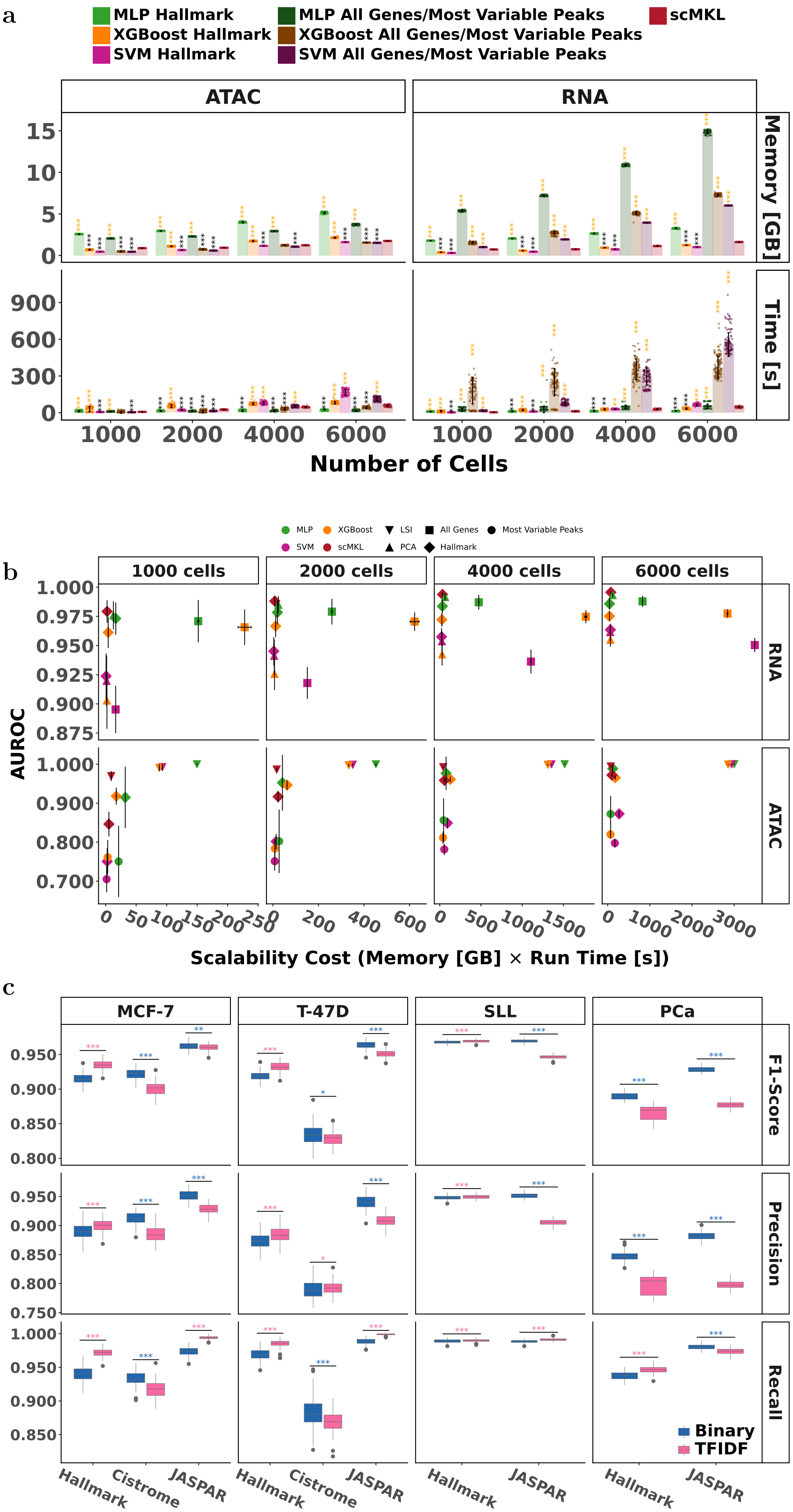
a
MLP Hallmark
XGBoost Hallmark
SVM Hallmark
MLP All Genes/Most Variable Peaks
XGBoost All Genes/Most Variable Peaks
SVM All Genes/Most Variable Peaks
scMKL
ATAC
RNA
Memory [GB]
Time [s]
Number of Cells
b
MLP
XGBoost
LSI
All Genes
Most Variable Peaks
SVM
scMKL
PCA
Hallmark
1000 cells
2000 cells
4000 cells
6000 cells
AUROC
RNA
ATAC
Scalability Cost (Memory [GB] × Run Time [s])
c
MCF-7
T-47D
SLL
PCa
F1-Score
Precision
Recall
Binary
TFIDF
Hallmark
Cistrome
JASPAR

Figure S2. scMKL provides scalable, flexible, and accurate predictions across diverse datasets and identifies biologically relevant mechanisms through pathway/TF selection.

a. Training memory usage comparison: Analysis of training memory (in GB) and time (in seconds) usage across the four machine learning models. *, **, *** are used to denote statistical significance from Wilcoxon test p values < 0.05, 0.01, and 0.001 respectively. Gold indicates scMKL had significantly better performance; black indicates the benchmark algorithm had significantly higher performance. **b.** Comparison of scalability cost including dimensionality reduction methods, PCA for RNA and LSI for ATAC: Analysis of training time in seconds and memory in GB across different sample sizes (1k-6k cells) for all the four machine learning models (MLP, XGBoost, SVM, and scMKL) using all genes, Hallmark genes, Hallmark peaks, and most variable peaks. **c.** Comparison of ATAC data groupings and transformations: Predictive performance for binary and TF-IDF normalized ATAC peaks using Hallmark gene sets, Cistrome and JASPAR TFBS as prior information. *, **, and *** denote significance determined by Wilcoxon tests for p-values < 0.05, 0.01, and 0.001 indicating which ATAC data transformation had significantly improved performance. Error bars are calculated as the standard deviation of the data presented.

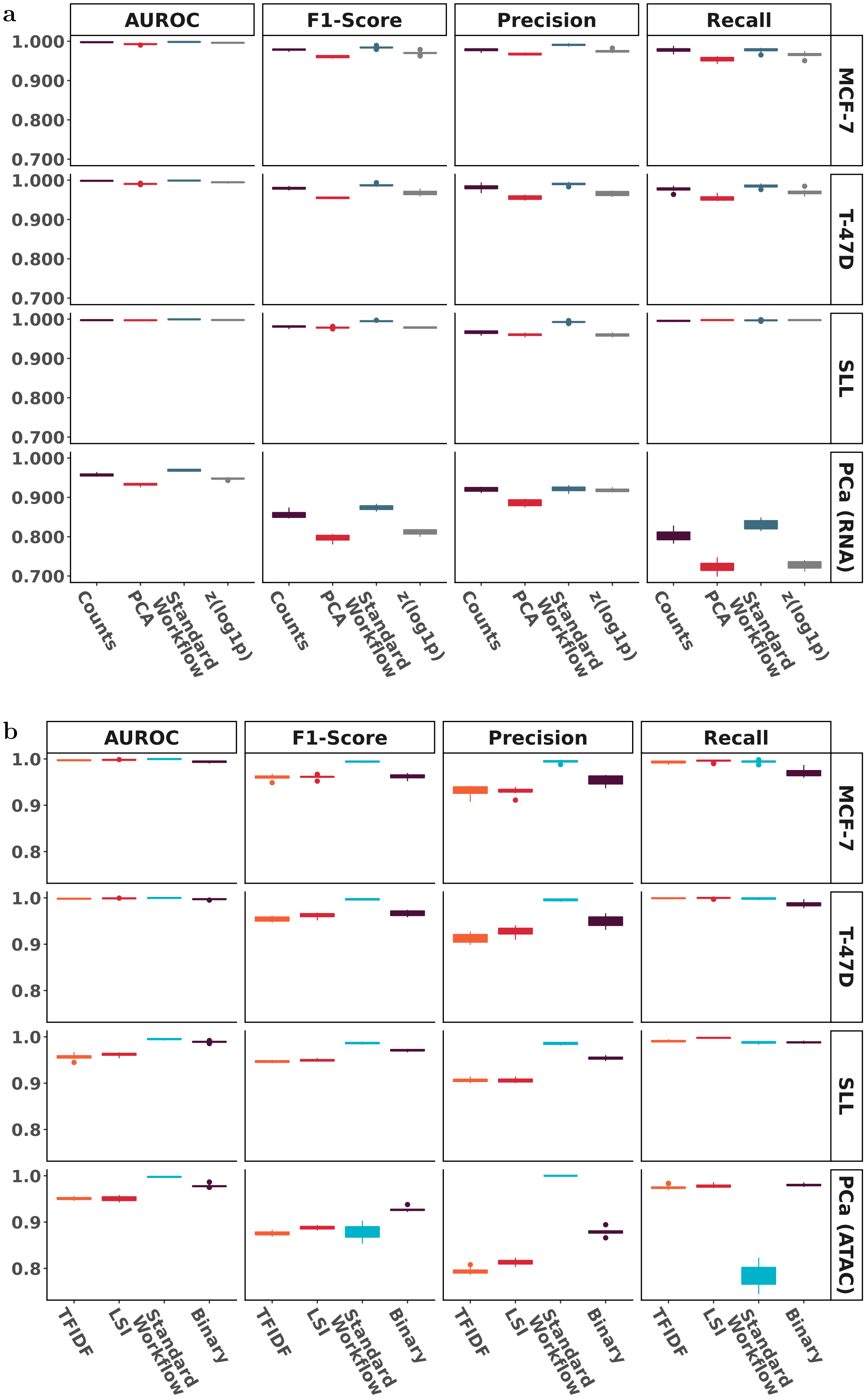
a
AUROC
F1-Score
Precision
Recall
MCF-7
T-47D
SLL
PCa (RNA)
1.000
0.900
0.800
0.700
Counts
PCA
Standard Workflow
z(log1p)
b
AUROC
F1-Score
Precision
Recall
MCF-7
T-47D
SLL
PCa (ATAC)
1.0
0.9
0.8
TFIDF
LSI
Standard Workflow
Binary

Figure S3. Comparison of preprocessing strategies for RNA and ATAC feature representations across multiple datasets.

a. RNA modality: AUROC, F1-score, precision, and recall for scMKL trained on RNA data from MCF7, T47D, SLL, and prostate cancer datasets. Comparisons include: PCA on log-normalized and z-scored RNA counts (50 components), standard workflow using all genes, and log(z)-transformed counts without dimensionality reduction. **b.** ATAC modality: AUROC, F1-score, precision, and recall for classifiers using ATAC peak counts from the same datasets. Comparisons include binary peak presence/absence matrix, TF-IDF transformation, and LSI via TruncatedSVD (50 components, first component removed), as well as the standard workflow using most variable peaks.

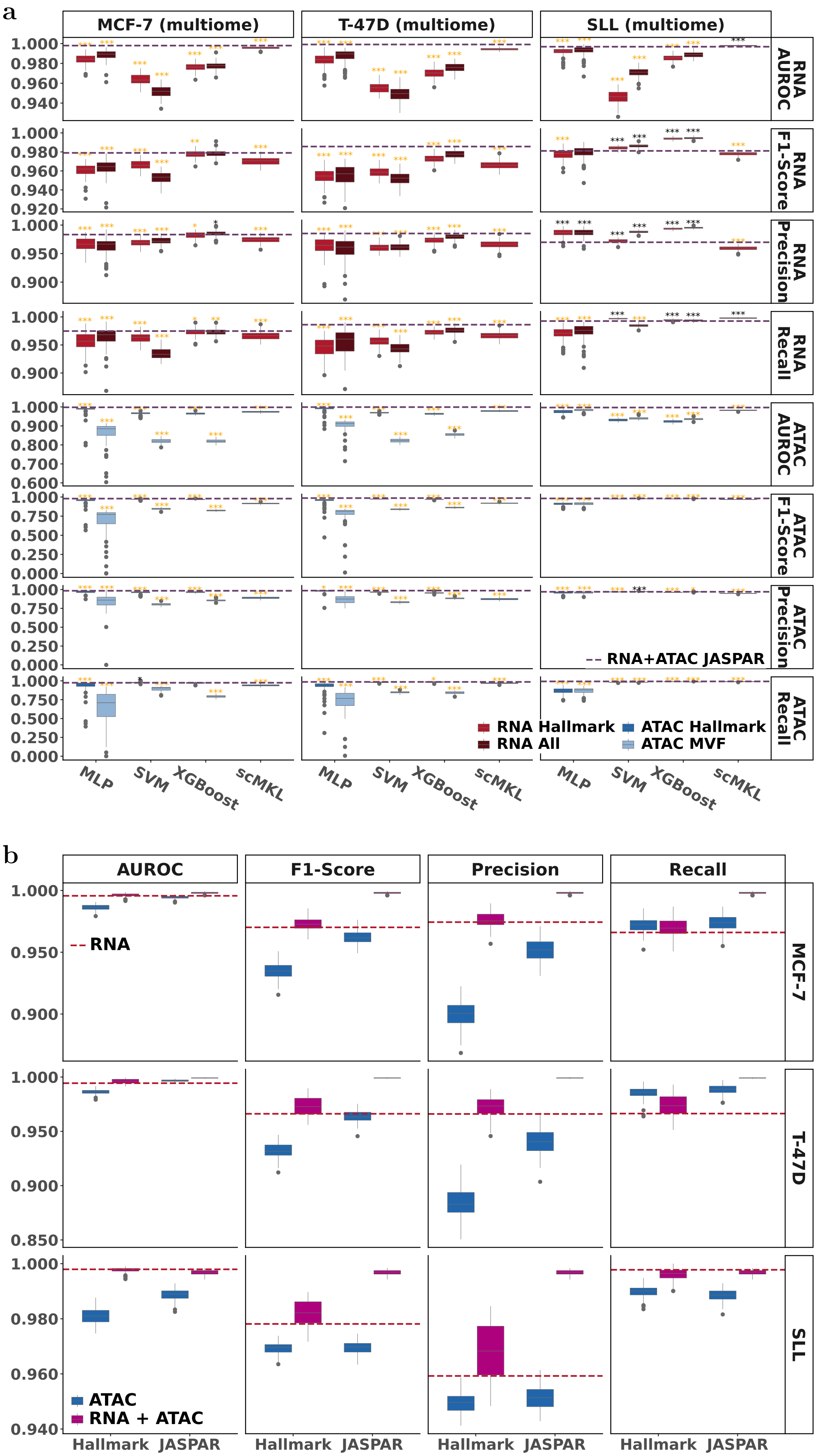
a
MCF-7 (multiome)
T-47D (multiome)
SLL (multiome)
RNA AUROC
RNA F1-Score
RNA Precision
RNA Recall
ATAC AUROC
ATAC F1-Score
ATAC Precision
ATAC Recall
RNA+ATAC JASPAR
RNA Hallmark
RNA All
ATAC Hallmark
ATAC MVF
MLP
SVM
XGBoost
scMKL
b
AUROC
F1-Score
Precision
Recall
RNA
MCF-7
T-47D
SLL
ATAC
RNA + ATAC
Hallmark
JASPAR

Figure S4. Biologically informed multimodal integration outperforms single-modality and state-of-the-art methods across diverse datasets.

a. Multimodal integration of RNA and ATAC using Hallmark gene set-informed gene groups and JASPAR TFBS-informed peak groups consistently outperforms state-of-the-art algorithms across all datasets. *, **, *** are used to denote statistical significance from Wilcoxon test p values < 0.05, 0.01, and 0.001 respectively. Gold indicates scMKL had significantly better performance; black indicates the benchmark algorithm had significantly higher performance. **b.** Multimodal integration of RNA and ATAC using JASPAR-informed features not only surpasses single-modality predictions but also outperforms Hallmark-based multimodal integration across all datasets and evaluation metrics.

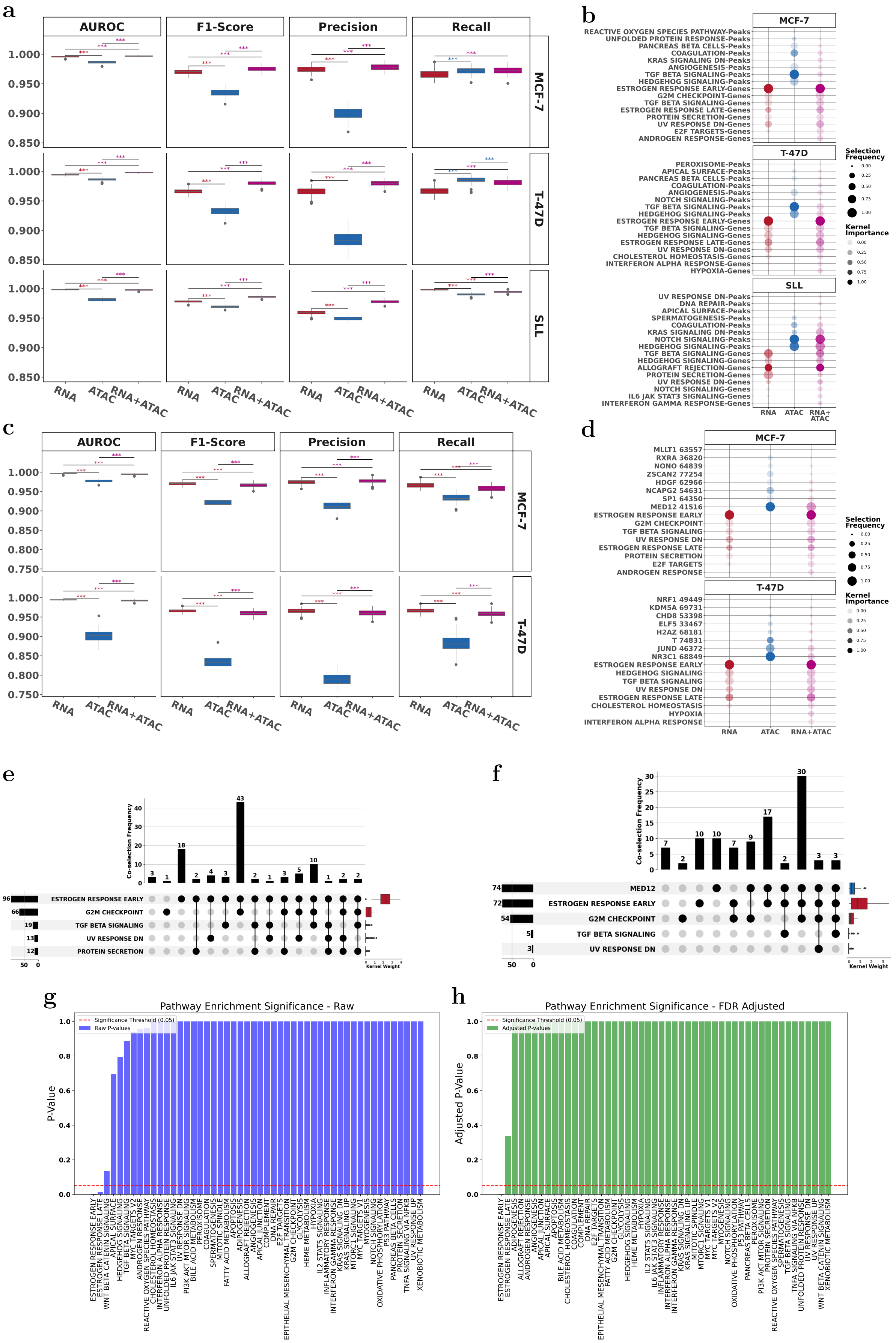

a
AUROC
F1-Score
Precision
Recall
MCF-7
T-47D
SLL
RNA
ATAC
RNA+ATAC
b
MCF-7
REACTIVE OXYGEN SPECIES PATHWAY-Peaks
UNFOLDED PROTEIN RESPONSE-Peaks
PANCREAS BETA CELLS-Peaks
COAGULATION-Peaks
KRAS SIGNALING DN-Peaks
ANGIOGENESIS-Peaks
TGF BETA SIGNALING-Peaks
HEDGEHOG SIGNALING-Peaks
ESTROGEN RESPONSE EARLY-Genes
G2M CHECKPOINT-Genes
TGF BETA SIGNALING-Genes
ESTROGEN RESPONSE LATE-Genes
PROTEIN SECRETION-Genes
UV RESPONSE DN-Genes
E2F TARGETS-Genes
ANDROGEN RESPONSE-Genes
T-47D
PEROXISOME-Peaks
APICAL SURFACE-Peaks
PANCREAS BETA CELLS-Peaks
COAGULATION-Peaks
ANGIOGENESIS-Peaks
NOTCH SIGNALING-Peaks
TGF BETA SIGNALING-Peaks
HEDGEHOG SIGNALING-Peaks
ESTROGEN RESPONSE EARLY-Genes
TGF BETA SIGNALING-Genes
HEDGEHOG SIGNALING-Genes
ESTROGEN RESPONSE LATE-Genes
UV RESPONSE DN-Genes
CHOLESTEROL HOMEOSTASIS-Genes
INTERFERON ALPHA RESPONSE-Genes
HYPOXIA-Genes
SLL
UV RESPONSE DN-Peaks
DNA REPAIR-Peaks
APICAL SURFACE-Peaks
SPERMATOGENESIS-Peaks
COAGULATION-Peaks
KRAS SIGNALING DN-Peaks
NOTCH SIGNALING-Peaks
HEDGEHOG SIGNALING-Peaks
TGF BETA SIGNALING-Genes
HEDGEHOG SIGNALING-Genes
ALLOGRAFT REJECTION-Genes
PROTEIN SECRETION-Genes
UV RESPONSE DN-Genes
NOTCH SIGNALING-Genes
IL6 JAK STAT3 SIGNALING-Genes
INTERFERON GAMMA RESPONSE-Genes
RNA
ATAC
RNA+ATAC
Selection Frequency
Kernel Importance
c
AUROC
F1-Score
Precision
Recall
MCF-7
T-47D
d
MCF-7
MLLT1 63557
RXRA 36820
NONO 64839
ZSCAN2 77254
HDGF 62966
NCAPG2 54631
SP1 64350
MED12 41516
ESTROGEN RESPONSE EARLY
G2M CHECKPOINT
TGF BETA SIGNALING
UV RESPONSE DN
ESTROGEN RESPONSE LATE
PROTEIN SECRETION
E2F TARGETS
ANDROGEN RESPONSE
T-47D
NRF1 49449
KDM5A 69731
CHD8 53398
ELF5 33467
H2AZ 68181
T 74831
JUND 46372
NR3C1 68849
ESTROGEN RESPONSE EARLY
HEDGEHOG SIGNALING
TGF BETA SIGNALING
UV RESPONSE DN
ESTROGEN RESPONSE LATE
CHOLESTEROL HOMEOSTASIS
HYPOXIA
INTERFERON ALPHA RESPONSE
e
Co-selection Frequency
ESTROGEN RESPONSE EARLY
G2M CHECKPOINT
TGF BETA SIGNALING
UV RESPONSE DN
PROTEIN SECRETION
Kernel Weight
f
Co-selection Frequency
MED12
ESTROGEN RESPONSE EARLY
G2M CHECKPOINT
TGF BETA SIGNALING
UV RESPONSE DN
Kernel Weight
g
Pathway Enrichment Significance - Raw
Significance Threshold (0.05)
Raw P-values
P-Value
h
Pathway Enrichment Significance - FDR Adjusted
Significance Threshold (0.05)
Adjusted P-values
Adjusted P-Value

Figure S5. Hallmark pathways and Cistrome TFs in the RNA+ATAC multimodal setting.

a. Hallmark pathways as prior information. Comparison of classification performance (AUROC, F1-score, precision, recall) between RNA (red), ATAC (blue), and RNA+ATAC (purple) models across MCF-7, T-47D, and SLL multimodal datasets. *, **, and *** denote significance determined by Wilcoxon tests for p-values < 0.05, 0.01, and 0.001 indicating which experiments resulted had significantly improved performance The color indicates which experiment had significantly higher performance **b.** Hallmark pathways as prior information. Feature group selection frequency and kernel importance scores across RNA, ATAC, and RNA+ATAC models, highlighting consistently selected biological pathways. **c.** Hallmark pathways and Cistrome TF as prior information. Comparison of classification performance (AUROC, F1-score, precision, recall) between RNA (red), ATAC (blue), and RNA+ATAC (purple) models across MCF-7 and T-47D multimodal datasets. *, **, and *** denote significance determined by Wilcoxon tests for p-values < 0.05, 0.01, and 0.001 respectively. The color indicates which experiment had significantly higher performance. **d.** Hallmark pathways and Cistrome TF as prior information. Feature group selection frequency and kernel importance scores across RNA, ATAC, and RNA+ATAC models, highlighting consistently selected biological pathways and TFs. **e.** Co-selection of Hallmark pathways in the RNA unimodal setting, demonstrating the independent selection ER Early pathway and its combined selections, while the ER Early pathway was consistently prioritized with a higher weight. **f.** In the RNA Hallmark + ATAC Cistrome multimodal setting, ER Early pathway and MED12 TF were selected independently and together with G2M pathway, while Estrogen Response g-h. Intersection of MED12-regulated regions with Hallmark pathway gene regions, showing a statistically significant overlap exclusively with the ER Early pathway.

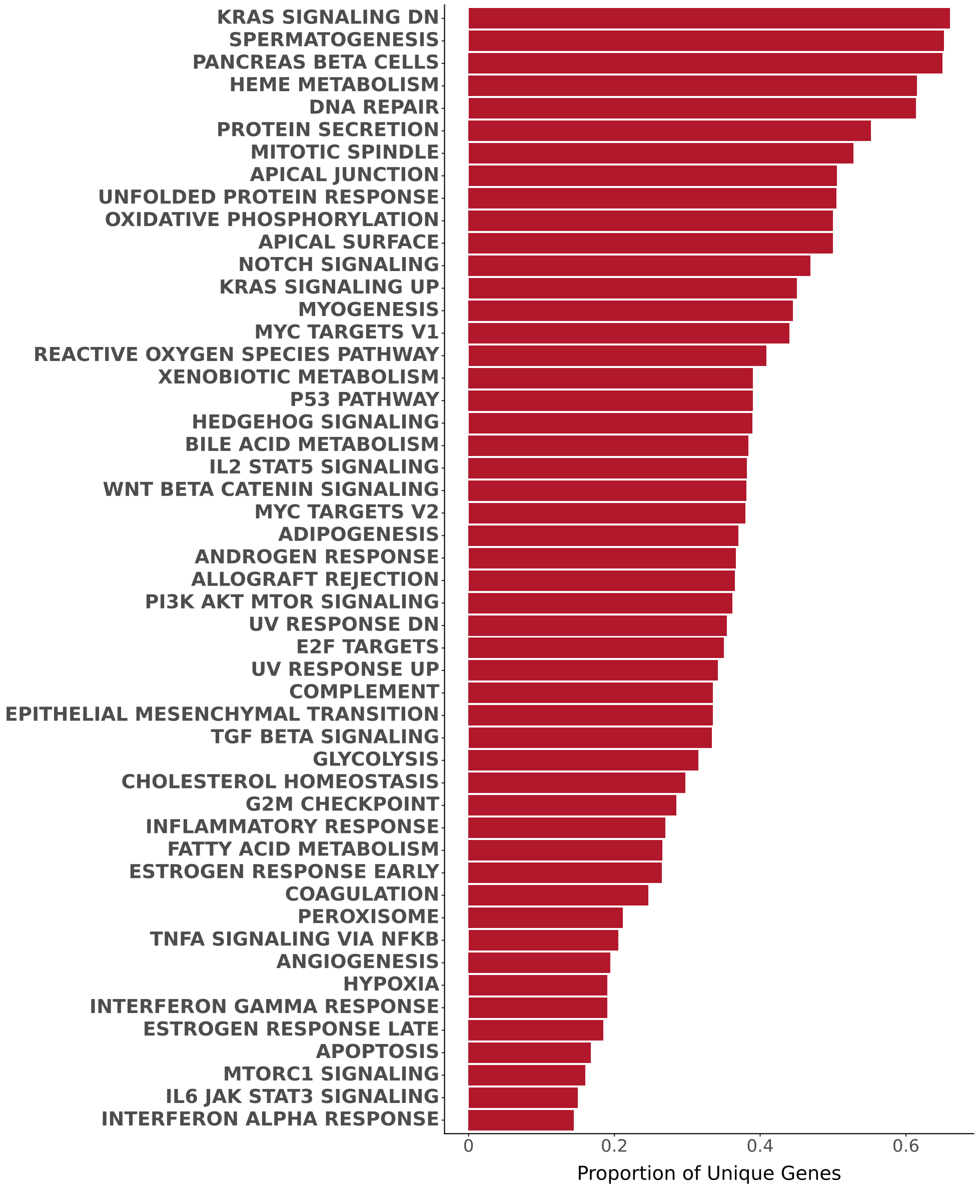

Figure S6. Hallmark pathways' predictive power is more than the sum of their genes'.

Proportion of genes which are unique to a given pathway.

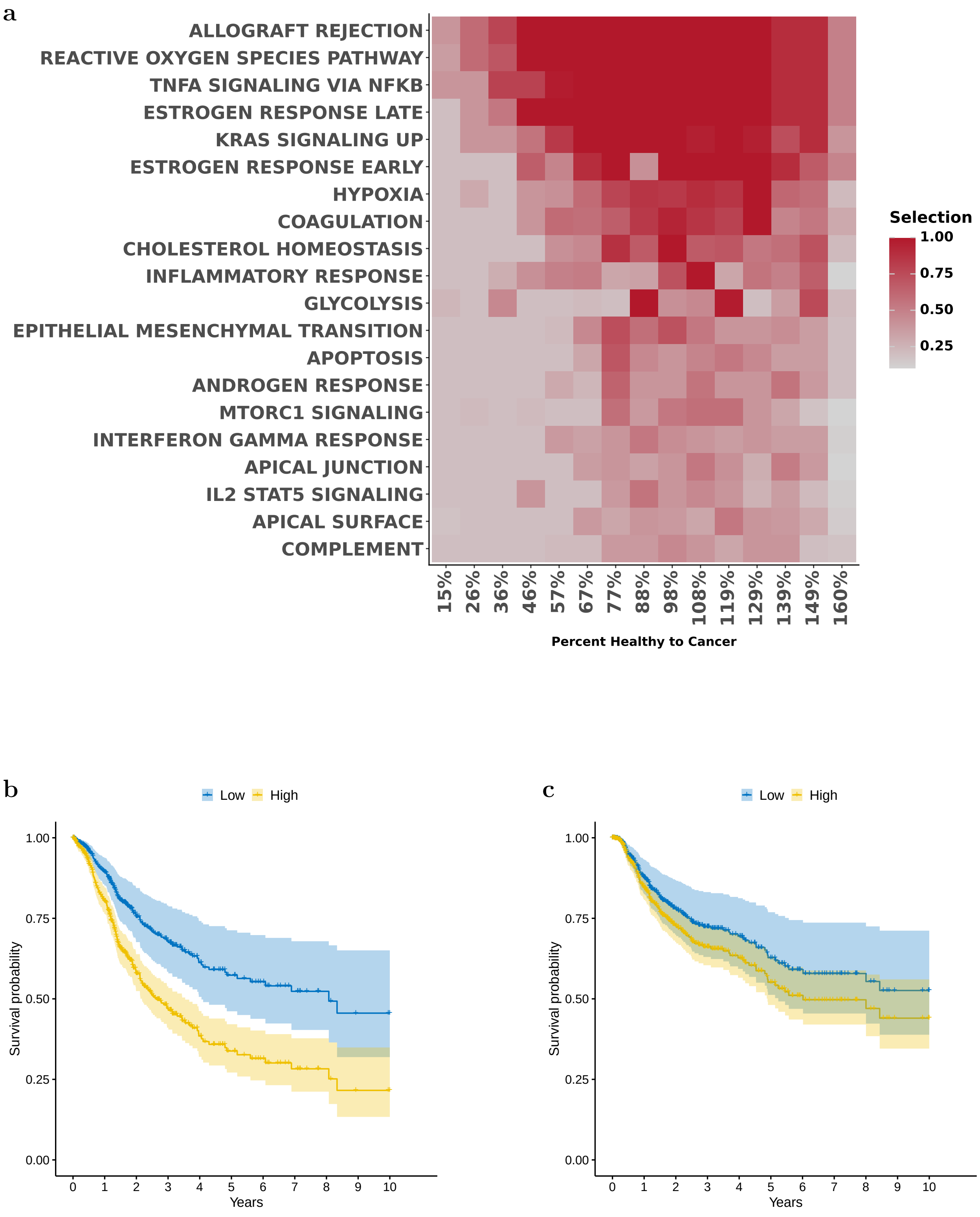

COX HALLMARK ER LATE LUAD

COX HALLMARK ER LATE LUSC

Figure S7. Related to main Figure 6 in the main text.

a. Overall pathway selection with varying class imbalance **b-c.** Survival analysis validating that scMKL finding, Hallmark ER Late pathway is LUAD-specific. Differential progression-free interval (PFI) analysis using a Cox proportional-hazards (PH) model for the Hallmark Estrogen Response Late pathway. Left: LUAD cohort (Hazard-ratio: 1.95, p-value <0.01). Right: LUSC cohort (Hazard ratio: 1.28, p-value 0.29). Hazard ratios and p-values reflect the association between pathway activity and patient outcome.

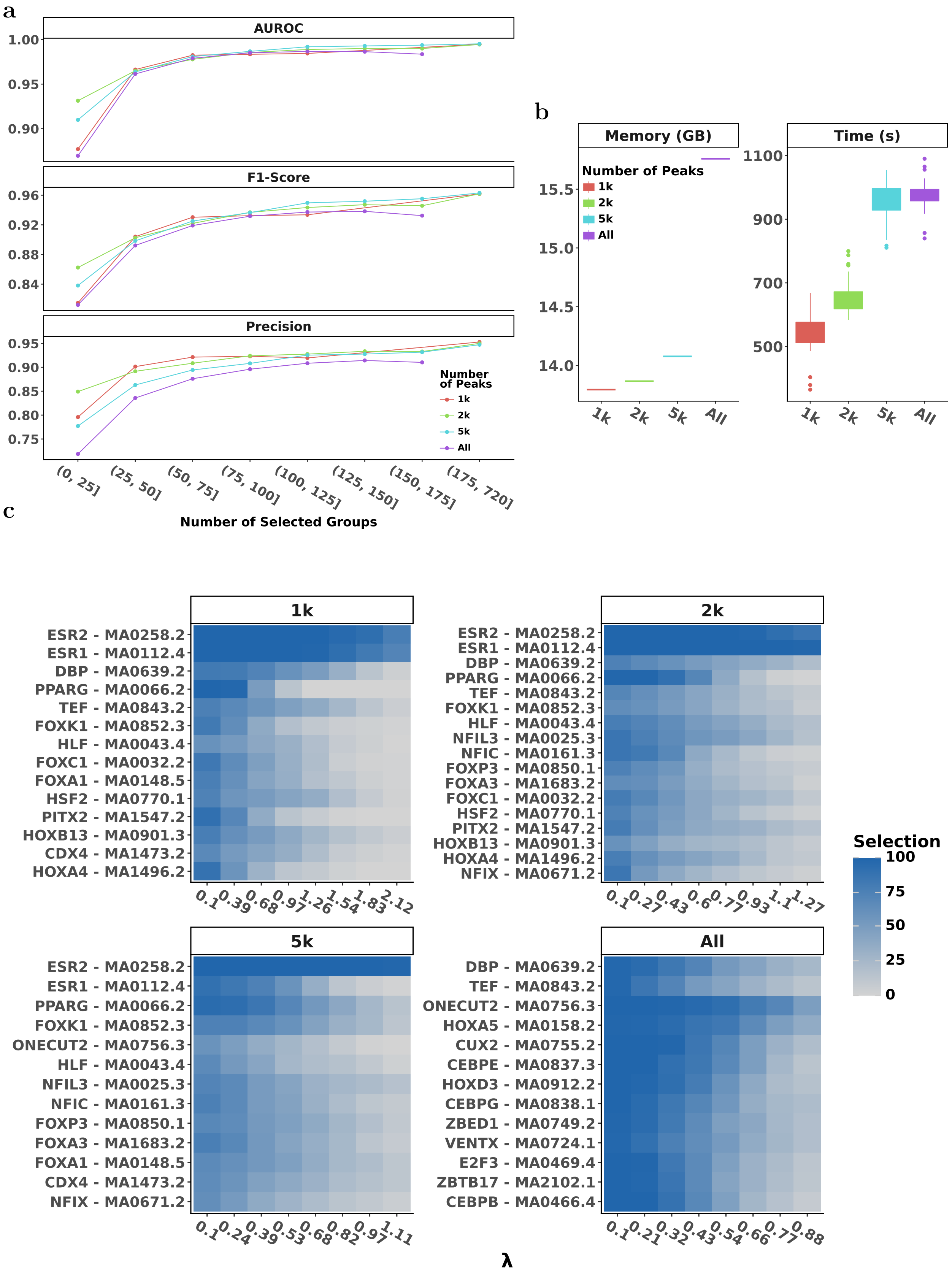
a
AUROC
1.00
0.95
0.90
F1-Score
0.96
0.92
0.88
0.84
Precision
0.95
0.90
0.85
0.80
0.75
Number of Peaks
1k
2k
5k
All
(0, 25]
(25, 50]
(50, 75]
(75, 100]
(100, 125]
(125, 150]
(150, 175]
(175, 720]
Number of Selected Groups
b
Memory (GB)
Time (s)
Number of Peaks
15.5
15.0
14.5
14.0
1100
900
700
500
c
1k
ESR2 - MA0258.2
ESR1 - MA0112.4
DBP - MA0639.2
PPARG - MA0066.2
TEF - MA0843.2
FOXK1 - MA0852.3
HLF - MA0043.4
FOXC1 - MA0032.2
FOXA1 - MA0148.5
HSF2 - MA0770.1
PITX2 - MA1547.2
HOXB13 - MA0901.3
CDX4 - MA1473.2
HOXA4 - MA1496.2
0.1 0.39 0.68 0.97 1.26 1.54 1.83 2.12
2k
ESR2 - MA0258.2
ESR1 - MA0112.4
DBP - MA0639.2
PPARG - MA0066.2
TEF - MA0843.2
FOXK1 - MA0852.3
HLF - MA0043.4
NFIL3 - MA0025.3
NFIC - MA0161.3
FOXP3 - MA0850.1
FOXA3 - MA1683.2
FOXC1 - MA0032.2
HSF2 - MA0770.1
PITX2 - MA1547.2
HOXB13 - MA0901.3
HOXA4 - MA1496.2
NFIX - MA0671.2
0.1 0.27 0.43 0.6 0.77 0.93 1.1 1.27
Selection
100
75
50
25
0
5k
ESR2 - MA0258.2
ESR1 - MA0112.4
PPARG - MA0066.2
FOXK1 - MA0852.3
ONECUT2 - MA0756.3
HLF - MA0043.4
NFIL3 - MA0025.3
NFIC - MA0161.3
FOXP3 - MA0850.1
FOXA3 - MA1683.2
FOXA1 - MA0148.5
CDX4 - MA1473.2
NFIX - MA0671.2
0.1 0.24 0.39 0.53 0.68 0.82 0.97 1.11
All
DBP - MA0639.2
TEF - MA0843.2
ONECUT2 - MA0756.3
HOXA5 - MA0158.2
CUX2 - MA0755.2
CEBPE - MA0837.3
HOXD3 - MA0912.2
CEBPG - MA0838.1
ZBED1 - MA0749.2
VENTX - MA0724.1
E2F3 - MA0469.4
ZBTB17 - MA2102.1
CEBPB - MA0466.4
0.1 0.21 0.32 0.43 0.54 0.66 0.77 0.88
λ

Figure S8. TFBS-informed grouping strategy comparisons.

a. Classification accuracy comparison of scMKL using MCF-7 ATAC data across four versions of TFBS-informed peak sets: top 1K, 2K, 5K most significant peaks, and all. Matched peaks from the JASPAR 2024 database across different regularization levels λ. **b.** Memory and time usage for each peak grouping strategy, showing that limiting peak sets (1k and 2k especially) substantially reduces computational cost while maintaining or improving performance. **c.** Heatmaps showing selected TF groups across different regularization (λ). Overlap in selected TFs across versions, emphasizing that high-confidence peak selection preserves core regulatory signals.

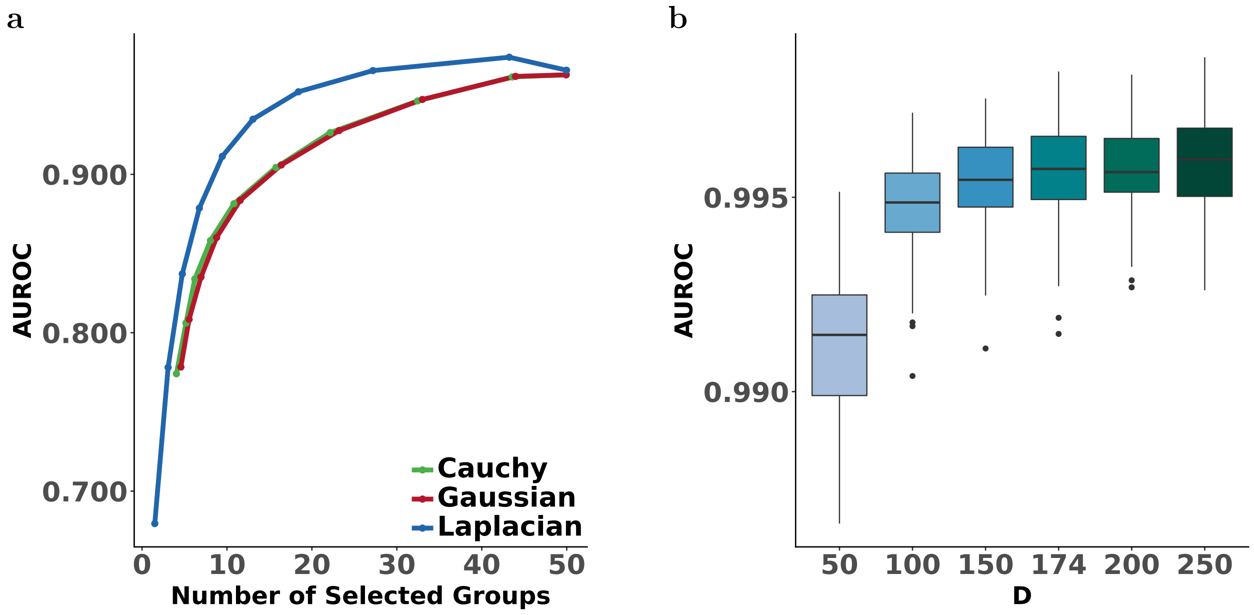

Figure S9. Optimization of kernel function and RFF dimensionality in scMKL.

a. AUROC performance of scMKL on MCF-7 ATAC data across different kernel functions (Cauchy, Gaussian, and Laplacian) using Hallmark pathway-based feature groupings at different solution sparsity. This comparison highlights the impact of kernel choice on model accuracy. **b.** AUROC as a function of RFF dimensionality (D) on MCF-7 RNA data, using Hallmark gene sets as prior feature groupings. Optimal performance occurs at intermediate D values, balancing accuracy and computational cost.

Table S1: Abbreviations and Definitions.

Abbreviation	Definition
AR	Androgen Response
ATAC	Assay for Transposase-Accessible Chromatin
AUROC	Area Under the Receiver Operating Characteristic curve
DEG	Differentially Expressed Gene
ER	Estrogen Response
GAS	Gene Accessibility Score
GL	Group Lasso
GREAT	Genomic Regions Enrichment of Annotations Tool
GSEA	Gene Set Enrichment Analysis
LUAD	Lung Adenocarcinoma
LUSC	Lung Squamous Cell Carcinoma
LSI	Latent Semantic Indexing
MKL	Multiple Kernel Learning
MLP	Multi-layer Perceptron
NE	Neuroendocrine
NSCLC	Non-Small Cell Lung Cancer
PCA	Principle Component Analysis
PCa	Prostate Cancer
RFF	Random Fourier Features
RNA-seq	Sequencing of Messenger Ribonucleic Acid
sc	Single-Cell
scMKL	Single-Cell Multiple Kernel Learning
SLL	Small Lymphocytic Lymphoma
TCGA	Tumor Cancer Genome Atlas
TF	Transcription Factor
TFBS	Transcription Factor Binding Sites
TF-IDF	Term Frequency-Inverse Document Frequency
UMAP	Uniform Manifold Approximation and Projection
