# Supplementary material for: Interpretable and integrative analysis of single-cell multiomics with scMKL
Source: Commun Biol. 2025 Aug 6;8:1160. doi: 10.1038/s42003-025-08533-7 (PMC12328712; doi:10.1038/s42003-025-08533-7)
Supplement: Supplementary file 2 — Reporting Summary [file 42003_2025_8533_MOESM2_ESM.pdf]

nature portfolio

Corresponding author(s): Cigdem Ak

Last updated by author(s): Jul 1, 2025

Reporting Summary

Nature Portfolio wishes to improve the reproducibility of the work that we publish. This form provides structure for consistency and transparency in reporting. For further information on Nature Portfolio policies, see our Editorial Policies and the Editorial Policy Checklist.

Statistics

For all statistical analyses, confirm that the following items are present in the figure legend, table legend, main text, or Methods section.

n/a	Confirmed	
☐	☒	The exact sample size (n) for each experimental group/condition, given as a discrete number and unit of measurement
☒	☐	A statement on whether measurements were taken from distinct samples or whether the same sample was measured repeatedly
☐	☒	The statistical test(s) used AND whether they are one- or two-sided<br>*Only common tests should be described solely by name; describe more complex techniques in the Methods section.*
☐	☒	A description of all covariates tested
☐	☒	A description of any assumptions or corrections, such as tests of normality and adjustment for multiple comparisons
☐	☒	A full description of the statistical parameters including central tendency (e.g. means) or other basic estimates (e.g. regression coefficient) AND variation (e.g. standard deviation) or associated estimates of uncertainty (e.g. confidence intervals)
☐	☒	For null hypothesis testing, the test statistic (e.g. F, t, r) with confidence intervals, effect sizes, degrees of freedom and P value noted<br>*Give P values as exact values whenever suitable.*
☒	☐	For Bayesian analysis, information on the choice of priors and Markov chain Monte Carlo settings
☒	☐	For hierarchical and complex designs, identification of the appropriate level for tests and full reporting of outcomes
☒	☐	Estimates of effect sizes (e.g. Cohen's d, Pearson's r), indicating how they were calculated

Our web collection on statistics for biologists contains articles on many of the points above.

Software and code

Policy information about availability of computer code

Data collection

No software was used.

Data analysis

The code used to generate data with scMKL is available on GitHub, and zenodo (https://github.com/ohsu-cedar-comp-hub/scMKL, 10.5281/zenodo.15397923).

The following software packages and versions were used:

For comparison to standard single-cell analysis workflows:
scanpy 1.10.1
muon 0.1.6
gseapy 1.1.3

For comparison to state-of-the-art supervised algorithms:
scikit-learn 1.5.0
keras 2.15.0
tensorflow 2.15.0
mklpy 0.6

For generating figures in the manuscript:
plotly 5.9.0
plotnine 0.12.1

umap-learn 0.5.6

For manuscripts utilizing custom algorithms or software that are central to the research but not yet described in published literature, software must be made available to editors and reviewers. We strongly encourage code deposition in a community repository (e.g. GitHub). See the Nature Portfolio guidelines for submitting code & software for further information.

Data

Policy information about availability of data

All manuscripts must include a data availability statement. This statement should provide the following information, where applicable:

- Accession codes, unique identifiers, or web links for publicly available datasets
- A description of any restrictions on data availability
- For clinical datasets or third party data, please ensure that the statement adheres to our policy

We analyzed 5 different single-cell cancer datasets across different technology platforms. The multiome data on breast cancer cell lines, MCF-7 and T-47D is available under the accession number GSE154873. Source data files for SLL multiome data are available on the 10x genomics website as 'Flash-Frozen Lymph Node with B Cell Lymphoma at https://www.10xgenomics.com/resources/datasets/. The raw single-cell ATAC-sequencing files and processed data files on prostate cancer are available under the GSE accession number: GSE171559. The raw single-cell RNA-sequencing FASTQ files and gene expression matrices files on prostate cancer are available in the GSE accession number: GSE176031. The raw NSCLC data can be found on the gene expression omnibus using accession numbers GSE136246 and GSE127465. Additionally, the Seurat object with cell annotations for both datasets can be found on figshare (https://figshare.com/collections/_/6222221).

Human research participants

Policy information about studies involving human research participants and Sex and Gender in Research.

Reporting on sex and gender	Both prostate cancer data obtained from men while no gender information was available for the lymphatic cancer data on 10X website. Both NSCLC datasets contained samples from men and women.
Population characteristics	For prostate cancer single-cell ATAC sequencing data, samples were obtained from men with prostate cancer diagnosed in years 2004-2019 and who were treated with radical prostatectomy. Subjects age ranged from 46-81 years (median 64 years). For prostate cancer single-cell RNA-sequencing data, samples were collected from primary prostate cancer patients. No information is available for the lymphatic cancer data on 10X website.The first NSCLC dataset (GSE136246) had patients ranging from 48-83 years. The second (GSE127465) contained samples from patients 61-83 years.
Recruitment	For prostate cancer single-cell ATAC-sequencing data: Samples were accrued with informed consent either through the (1) OHSU Knight Cancer Institute (KCI) BioLibrary; a tissue repository (OHSU IRB#4918) that provides deidentified human biospecimens and associated metadata, or (2) a separate study (OHSU IRB#18321) designed to investigate prostate cancer MRI, histology findings, and molecular characterization of tumors among men undergoing radical prostatectomy (PI Kopp). Patients were not compensated for participation in both studies. In both of these settings, prostate cancer samples were provided from men who underwent radical prostatectomy thus elected and/or were selected for surgery; thus there is potentially some bias towards younger, healthier men with prostate cancer compared to men who undergo radiotherapy. However, the oldest man represented was age 81. There may also be a bias towards larger, more clinically significant tumors among the lower risk group as these men were not selected for active surveillance.<br><br>For prostate cancer single-cell RNA-sequencing data, eleven localized prostate cancer patients receiving urologic care were enrolled for single-cell RNA sequencing. Biopsy samples were collected from six prostate regions across three patients, while four patients provided tumor-only samples from radical prostatectomies (RP), and four provided both tumor and matched normal samples from RP. All RP patients had preoperative MRI or ultrasound-visible lesions, which were pathologically confirmed as cancer.<br><br>No information is available for the lymphatic cancer data on 10X website.<br><br>All NSCLC samples resulted from a variety of surgical procedures. For more information, see the original studies.
Ethics oversight	For single-cell ATAC-seq, oversight was provided by the OHSU IRB 4918 and 1821. For sc-RNA-seq, the UCSF Institutional Review Board (IRB) committee approved the collection of these patient data. The lung studies were conducted with approval of the Dana-Farber Brigham and Women's Cancer Center IRB and written informed consent from subjects.

Note that full information on the approval of the study protocol must also be provided in the manuscript.

Field-specific reporting

Please select the one below that is the best fit for your research. If you are not sure, read the appropriate sections before making your selection.

☒ Life sciences ☐ Behavioural & social sciences ☐ Ecological, evolutionary & environmental sciences

For a reference copy of the document with all sections, see nature.com/documents/nr-reporting-summary-flat.pdf

Life sciences study design

All studies must disclose on these points even when the disclosure is negative.

Sample size	For single-cell ATAC-sequencing data on prostate cancer, hundreds of individual profiles were obtained from similar size tumors. All patients meeting the clinical criteria with proper consent were included. We included at least seven patients of each clinical grade: primary Gleason pattern 3 (8 patient samples) and primary Gleason pattern6 4 (7 patient samples). Same sizes were selected to be equal across clinical grades to enable proper representation. A total of 12,717 single-cells were analyzed in this study.<br><br>For single-cell RNA-sequencing data om prostate cancer, eleven localized prostate cancer patients receiving urologic care were enrolled for single-cell RNA sequencing. Biopsy samples were collected from six prostate regions across three patients, while four patients provided tumor-only samples from radical prostatectomies (RP), and four provided both tumor and matched normal samples from RP. All RP patients had preoperative MRI or ultrasound-visible lesions, which were pathologically confirmed as cancer. 13,322 epithelial cells out of a total of 21,743 cells were analyzed in this study.<br><br>A total of 13,498 cells from lymphatic cancer, 6,438 cells from the MCF-7 breast cancer cell line, and 6,615 cells from the T-47D breast cancer cell line were analyzed using single-cell multiomics (RNA+ATAC) datasets. Our lung adenocarcinoma dataset contains 74,084 cells while the lung squamous cell carcinoma dataset contains 16,287 cells.<br><br>This study utilized publicly available single-cell multi-omics datasets. Given the nature of the study, the sample size was determined by the size of the available datasets, which were sufficiently large to ensure statistical power for machine learning model training and validation. We provided a detailed sample size description in Table 1.
Data exclusions	No cells or features were excluded unless they had zero variance (no change across samples), aside from applying the standard single-cell analysis workflow when comparing results.
Replication	To ensure the robustness and reproducibility of the scMKL model, we performed 100 independent replications of train-test splits, along with a holdout dataset used for final testing. This approach allowed us to assess model performance across multiple random splits and ensured consistent results. Additionally, all source code, data processing pipelines, and trained models are made available through GitHub to facilitate reproducibility. Single-cell multi-omics data, including scRNA-seq and scATAC-seq, are publicly accessible through GEO accession numbers reported.<br><br>For transfer learning runs with NSCLC, only 10 replications were run sampling different cells from the majority class each replication.
Randomization	Randomization is not applicable in this study, as the analysis was performed on pre-existing single-cell datasets.
Blinding	Blinding was not applicable in this study since all computational analyses were conducted on publicly available, deidentified single-cell datasets.

Reporting for specific materials, systems and methods

We require information from authors about some types of materials, experimental systems and methods used in many studies. Here, indicate whether each material, system or method listed is relevant to your study. If you are not sure if a list item applies to your research, read the appropriate section before selecting a response.

Materials & experimental systems

n/a	Involved in the study	
☒	☐	Antibodies
☐	☒	Eukaryotic cell lines
☒	☐	Palaeontology and archaeology
☒	☐	Animals and other organisms
☒	☐	Clinical data
☒	☐	Dual use research of concern

Methods

n/a	Involved in the study	
☒	☐	ChIP-seq
☒	☐	Flow cytometry
☒	☐	MRI-based neuroimaging

Eukaryotic cell lines

Policy information about cell lines and Sex and Gender in Research

Cell line source(s)	Both the MCF-7 and T-47D breast cancer cell lines are derived from female patients. Multiome data on breast cancer cell lines is available under the accession number GSE154873.
Authentication	N.A.
Mycoplasma contamination	N.A.

Commonly misidentified lines<br>(See ICLAC register)	N.A.
